# Numerical Investigation on Thermal Fluid Flow-Induced Porosity During EBW of Aluminum Alloy

**DOI:** 10.3390/ma19112233

**Published:** 2026-05-25

**Authors:** Ruchuan Zeng, Debin Song, Hongjian Cui, Ting Wang

**Affiliations:** 1 Beijing Spacecraft Manufacturing Co., Ltd., Beijing 100094, China; 2 State Key Laboratory of Precision Welding & Joining of Materials and Structures, Harbin Institute of Technology, Harbin 150001, China; 3 Shandong Provincial Key Laboratory of Special Welding Technology, Harbin Institute of Technology at Weihai, No. 2, West Wenhua Road, Weihai 264209, China

**Keywords:** electron beam welding, porosity, fluid flow, keyhole formation

## Abstract

This study proposes a novel dynamic composite heat source combining discrete tracking and vapor heating, which can precisely capture the transient energy deposition at the keyhole wall, and further discusses the formation mechanism of weld defects by investigating keyhole evolution and melt pool flow behavior. The weld morphology and dimensions predicted by the simulation are in good agreement with the experimental data, revealing the coupled mechanism between keyhole instability and porosity formation, as well as the generation mechanism of process-type porosity mainly influenced by the keyhole dynamic characteristics and the melt pool flow field together; specifically, keyhole instability forms a vapor cavity that will generate bubbles to participate in melt pool flow if it cannot be re-fused with the keyhole, and the bubble trajectory is related to buoyancy, gravity and liquid flow in the melt pool, with larger bubbles less likely to escape due to greater liquid viscous force. In addition, this study finds that increasing weld power and weld speed helps improve keyhole stability, weaken melt pool circulation intensity and shorten bubble escape path, thereby fundamentally revealing the formation mechanism of porosity defects during electron beam welding (EBW) of aluminum alloy, providing an effective numerical tool for optimizing EBW process parameters, and proposing corresponding inhibition measures to improve weld quality.

## 1. Introduction

High-strength aluminum alloys possess excellent mechanical and physical properties and have been widely used in industrial engineering [1,2,3]. Electron beam welding features low heat input, high power density and a large depth-to-width ratio, showing unique advantages in the welding of thick aluminum alloy plates [4,5]. However, the use of electron beam welding on thick aluminum alloy plates may result in process-induced porosity defects that can adversely affect the mechanical properties of the joint [6,7,8,9,10]. In electron beam welding, the interactions among heat transfer within the molten pool, liquid flow dynamics, and keyhole evolution are interdependent and collectively determine weld quality [11]. Therefore, research into the dynamics of the molten pool is essential for effectively controlling welding quality.

Researchers have adopted various experimental techniques to characterize the electron beam welding process, including CCD camera technology [12], X-ray imaging technology [13], and transparent material approaches [14]. Despite significant advancements in both theoretical analysis and experimental investigations regarding molten pool flow fields domestically and internationally, sensors are inadequate for effectively capturing transient changes in temperature fields and flow patterns within the molten pool. In contrast, numerical simulation methods present advantages, including low cost and high efficiency. Panwisawas et al. [15] simulated keyhole and pore formation during the laser welding of Ti-6Al-4V alloy, and concluded that process-induced porosity is mainly governed by plate thickness, laser power and welding speed. T. F. Flint [16] numerically studied the effects of electron beam divergence and focal position on the substrate. The results reveal that the beam focal location directly dominates keyhole dynamic behavior and morphological characteristics. A low-divergence electron beam achieves a larger penetration depth and maintains a more stable thermocapillary flow. Ke [17] investigated melting/solidification behavior, flow patterns, keyhole dynamics and energy absorption under keyhole, transition and conduction welding modes. Ye and Chen [18] simplified the keyhole as a cylindrical structure and analyzed molten pool heat transfer, as well as Marangoni-driven coupled flow. Duggirala [19] established a two-dimensional keyhole profile model by combining keyhole drilling velocity and welding speed.

In summary, scholars worldwide have achieved remarkable progress in the numerical simulation of molten pool flow fields. Nevertheless, conventional analytical heat source models are usually calibrated based on weld pool geometry, and their energy distribution deviates greatly from the actual thermal loading in electron beam welding, making it difficult to accurately reproduce molten pool flow characteristics. Although a few studies such as Liu [7] proposed a surface heat source that tracks the keyhole bottom, this method has evident limitations. Affected by the violent fluctuation of real keyholes, merely tracking the keyhole bottom cannot realize effective energy coupling between the electron beam and the keyhole free surface; meanwhile, this approach assumes an ideal invariant keyhole morphology. In this paper, a novel dynamic heat source model is proposed. Through numerical simulation and experimental verification, the effect of process parameters on the flow behavior of the molten pool during the electron beam welding of 5B70 aluminum alloy is analyzed, and the generation mechanism and influencing factors of process-type porosity defects are discussed. The innovation of the proposed model lies in constructing a composite heat source combining a discrete tracking heat source and a vapor heat source, which can more accurately characterize the energy distribution during electron beam welding. The tracking heat source considers the radial heat flux distribution of the electron beam and the energy attenuation induced by electron scattering in metal vapor along the penetration direction. The surface heat source is discretized to realize dynamic energy coupling between the electron beam and the keyhole wall. The vapor heat source adopts a Gaussian distribution to simulate the thermal effect of internal vapor on the keyhole wall.

## 2. Experiment and Numerical Implementations

The experimental material employed in this paper is the 5B70 aluminum alloy plate (Beijing Satellite Manufacturing Factory Co., Ltd., Beijing, China), and its chemical composition is presented in Table 1. The metallographic specimens were photographed, recorded, and observed using the Olympus DSX-510 optical digital microscope (Olympus Corporation, Tokyo, Japan). The numerical simulation of the electron beam inclined welding process of the aluminum alloy was conducted by means of the Fluent fluid dynamics software (Fluent 2020 R2). After the completion of the calculation, the results were analyzed using the CFD post-processing software (CFD-post 2020 R2).

### 2.1. Basic Assumptions and Governing Equations

#### 2.1.1. Basic Assumptions

**Figure 1:**
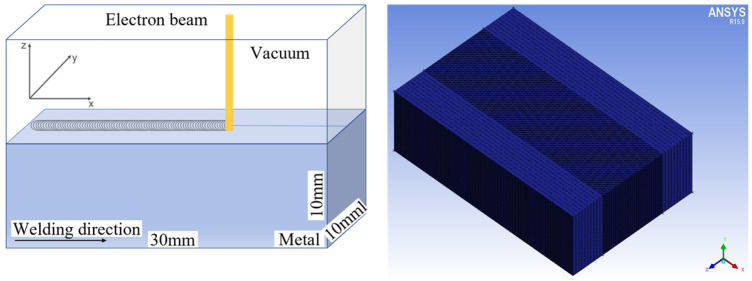
Computational domain for the simulations.

As illustrated in Figure 1, a geometric model for the calculation domain of electron beam welding is established. ICEM-CFD (2020 R2) software is utilized to create a three-dimensional geometric model and perform meshing. The computational domain is set to 30 mm × 10 mm × 10 mm, which fully covers the full penetration depth of the weld and a sufficiently wide heat-affected zone to eliminate boundary effects. The minimum grid size is set to 0.1 mm, which is adopted in the vicinity of the keyhole to accurately capture the sharp gradients of temperature and flow velocity. A fixed time step of 1 × 10^−6^ s is employed in the numerical calculation. The total number of grid nodes is 402,696, with a total grid cell count of 313,000. The residual convergence criterion is set to 10^−6^ for the continuity and momentum equations, and 10^−7^ for the energy equation. The second-order upwind scheme is applied for the discretization of the momentum and energy equations, while the SIMPLE algorithm is adopted for pressure–velocity coupling in Fluent. The molten pool flow is assumed to be laminar. To simplify the numerical calculation, several basic assumptions as follows are made in this study:

(1) The initial working temperature is defined as 300 K, with an air pressure of 0 Pa in the vacuum region.

(2) The molten metal within the weld pool is treated as an incompressible Newtonian fluid.

(3) Mass loss resulting from vaporization of metal on the surface of the molten pool is disregarded.

(4) Specific heat capacity, thermal conductivity, and density are considered functions of temperature; other material properties remain constant.

#### 2.1.2. Governing Equations

In the computational analysis of the flow field, the equations governing energy conservation, momentum conservation, and mass conservation are rigorously satisfied within the computational domain. Additionally, the volume of fluid (VOF) method is employed to accurately trace the free surface of the keyhole. This method can precisely capture the sharp gas–liquid interface with low numerical diffusion, effectively suppressing interface blurring and numerical dissipation.

Energy conservation equation:

(1) $$\frac{\partial E}{\partial t}=\nabla (EV)+\nabla (K\nabla T)+q_{sor}$$

Momentum conservation equation:

(2) $$\frac{\partial V}{\partial t}=-v(v\nabla )+\mu \nabla^{2}v-\frac{1}{\rho }\nabla p+\frac{1}{\rho }F_{SOR}$$

Mass conservation equation:

(3) $$\frac{\partial \rho }{\partial t}+\nabla •(\rho •\vec{v)}=R_{SOR}$$

Liquid–gas interface tracking equation:

(4) $$Vf•\frac{\partial \rho }{\partial t}+\nabla •(A•U)=0$$

where *E* is the internal energy of the material and *k* is the thermal conductivity of the material. The transient, convective, diffusive and source terms are shown from left to right. The source term is zero because the heat generation by viscous force is not considered. *p* denotes the fluid pressure. ρ is the density, $\vec{v}$ is the fluid velocity, and $R_{sor}$ is the mass source term. $R_{sor}$ is zero because the wire feed is not considered in this paper.

### 2.2. Boundary Conditions

These surfaces are treated as wall boundaries with symmetric boundary conditions. On the free boundary of the keyhole wall, two primary boundary conditions are applied: the momentum boundary condition and the energy boundary condition.

Buoyancy:

(5) $$S_{B}=\rho g\beta •(T-T_{1})$$

where  β is the coefficient of volume expansion of the metal and $T_{l}\;$ is the liquid phase line temperature.

Surface tension:

(6) $$F_{\sigma }=\sigma kn+\nabla \sigma =\sigma kn+\frac{\partial \sigma }{\partial t}\frac{dT}{dl}$$

(7) $$\sigma (T)=\sigma_{m}-\lambda (T-T_{m})$$

where σ is the surface tension coefficient; $\sigma_{m\;}$ is the surface tension of the material at the melting point $T_{m}$; γ is the gradient of the temperature coefficient of surface tension; and k is the surface curvature of the melt pool.

Metal vapor recoil pressure:

(8) $$F_{v}=\alpha •P_{0}•\exp\left[\frac{Μ•∆H_{vap}}{R}(\frac{1}{{Τ}_{b}}-\frac{1}{Τ})\right]$$

where α is the coefficient of the vapor reaction force of the metal, which takes the value of 0.54 in the model $P_{0}$ is the standard atmospheric pressure of 101,325 Pa; *M* is the molar mass of the metal; *R* is the gas constant, ∆*Hvap* is the heat of vaporization of the metal, and ${\;T}_{b}$ is the boiling point of the metal.

The heat loss due to metal evaporation and radiation is considered in the energy boundary conditions and is expressed as follows:

(9) $$-k\frac{\partial Τ}{\partial n}=q_{md}+q_{evap}=\varepsilon_{0}\sigma_{0}({Τ}^{4}-{Τ}_{0}^{4})+m_{v}H_{v}$$

where $\varepsilon_{0}$ is the surface radiation coefficient, $\sigma_{0}$ is Boltzmann’s constant *T*_0_ is the ambient temperature, $m_{v}$ is the rate of evaporation, and $H_{v}$ is the heat of vaporization of the metal.

## 3. Heat Source Model

**Figure 2:**
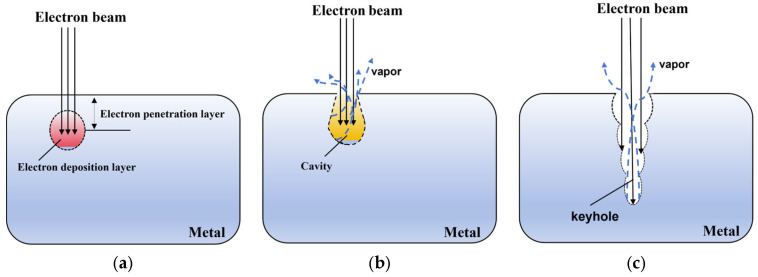
Schematic diagram of keyhole formation for deep-penetration EBW. (**a**) Energy deposition and heating stage (**b**) Melting and initial cavity formation stage (**c**) Stable keyhole formation stage.

When performing electron beam welding, the process primarily relies on the keyhole deep penetration effect. As illustrated in Figure 2, high-speed electrons penetrate through the metal surface to create an electron deposition layer. The metal within this energy deposition region reaches its boiling point in a very short time frame, resulting in vaporization and the formation of metal vapor. The presence of metal vapor exerts a dual influence. On the one hand, a significant pressure gradient exists between the high-concentration metal vapor within the keyhole and the ambient vacuum background. Driven by this gradient, metal vapor continuously ejects outwards from the keyhole, which breaks the surface molten layer of the base material and induces groove initiation. The intense scouring action at these groove openings further redistributes the molten metal flow, thereby facilitating the generation of typical weld nail-head features. On the other hand, the recoil pressure induced by metal vapor expels the molten liquid metal radially outward. Such material outflow exposes fresh solid metal surfaces and initially creates isolated elliptical cavities. Subsequent continuous energy deposition of the electron beam on the newly formed cavity bottom triggers repeated melting and cavity expansion. This cyclic process eventually evolves into a chain of mutually interconnected elliptical cavities within the weld seam.

The characteristics of the electron beam welding principle are integrated into a proposed composite heat source model, which consists of a “vapor heat source + discrete tracking heat source”. The vapor heat source is employed to simulate the flushing effect produced by high-temperature metal vapor at the upper region of the keyhole. In this model, the electron beam is conceptualized as several discrete independent rays, with each ray’s energy being directly deposited onto the “top free surface”. This approach disregards any reflection of the electron beam and utilizes a fixed value for absorption rate. The heat source initially interacts with the metal surface, facilitating gradual melting from the surface inward through thermal conduction. It consistently influences the molten pool’s surface as its morphology evolves. This method presents a more realistic representation compared with earlier volumetric heat sources.

The radial energy distribution of the initial electron beam adopts the well-recognized Gaussian distribution. The electron beam consists of discrete high-energy electrons, and it is overly complicated to model each electron individually. In this work, the heat source is discretized into a series of rays along the normal direction.

**Figure 3:**
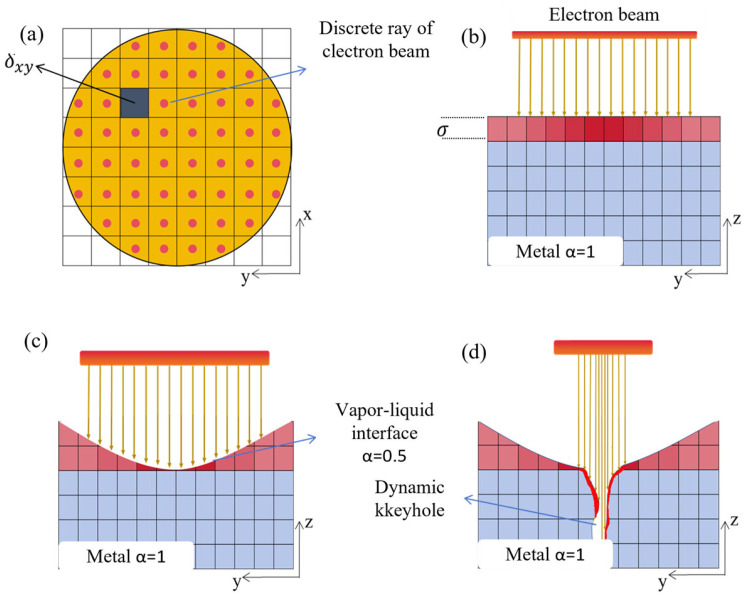
Schematic diagram of electron beam energy loading: (**a**) discretization of plane heat source; (**b**) ray energy deposition; (**c**) electron beam tracking of solid–liquid interface; (**d**) coupling between electron beam and keyhole wall.

The radial energy distribution of the initial electron beam adopts Gaussian distribution. The electron beam consists of discrete high-energy electrons, and it is overly complicated to model each electron individually. In this work, the heat source is discretized into a series of rays along the normal direction. The basic principle is described as follows. First, as shown in Figure 3a, the surface heat source is discretized into several rays with a diameter of δ_xy_. Then, relying on the user-defined function (UDF) in Fluent, the topmost interface unit with a volume fraction α ≥ 0.5 in each column is marked in sequence. Taking this position as the center, the grids within the depth σ below are further marked, where σ represents the energy deposition depth of the electron beam. Accordingly, each discretized energy ray is always loaded within the grids with a diameter of δ_xy_ and a depth of σ, as shown in Figure 3b,c. As the liquid metal is squeezed away by the recoil pressure, the thermal energy is continuously loaded on the keyhole wall, realizing the coupling between the heat source and the keyhole wall, as displayed in Figure 3d.

The inelastic scattering process is described using the relativistically modified Møller scattering cross section, which quantifies the probability of inelastic collisions between high-energy electrons and metal vapor atoms and is integrated over the energy loss range to obtain the effective cross section:

(10) $$\sigma_{inel}=\int_{\varepsilon_{i}}^{0.5}\frac{d\sigma }{d\varepsilon }d\varepsilon =\frac{2\pi e^{4}}{mv^{2}E_{0}}\cdot \left[\left(\frac{1}{2}-\varepsilon_{i}\right){\left(\frac{\tau }{1+\tau }\right)}^{2}+\frac{1}{\varepsilon_{i}}-\frac{1}{1-\varepsilon_{i}}-\frac{2\tau +1}{{\left(1+\tau \right)}^{2}}\ln\frac{1-\varepsilon_{i}}{\varepsilon_{i}}\right]$$

where τ is the normalized relativistic electron kinetic energy, m is the electron mass, and v is the relativistic velocity. γ is the relativistic correction factor.

Equations (11) and (12) present the relativistic Bethe formula for calculating the energy loss rate of electrons propagating in the material, where the energy-dependent term is defined by Equation (12).

(11) $$-\frac{dE}{ds}=\frac{2\pi e^{4}}{mv^{2}}nZ\left[\ln\frac{mv^{2}E}{2\gamma^{2}}-\left(2\lambda -\lambda^{2}\right)\ln2+\lambda^{2}+\frac{\left(1-\lambda^{2}\right)}{8}\right]$$

(12) $$E=mc^{2}\left(\lambda^{-1}-1\right)$$

where m is the electron mass and v is the relativistic velocity; c is the speed of light in vacuum; γ is the relativistic correction factor.

Combining the above factors, the expression for the electron beam heat source is established as:

(13) $$q_{1}(x,y)=\frac{3\eta_{1}\eta_{B}b^{2}UI}{\pi \sigma a^{2}\left(b^{2}+z^{2}\right)}\exp\left[-3\frac{b^{2}\left(x^{2}+y^{2}\right)}{a^{2}\left(b^{2}+z^{2}\right)}\right]$$

(14) $$q_{2}(x,y)=\frac{3\eta_{2}Q}{\pi r_{0}^{2}}\exp(-3\frac{x^{2}+y^{2}}{r_{0}^{2}})$$

where *q*_1_ is the keyhole tracking heat source and *q*_2_ is the vapor scouring heat source. *ŋ*_1_, *ŋ*_2_ are the welding efficiencies, which sum to 1, and the values of *ŋ*_1_, *ŋ*_2_ are 0.9 and 0.1, respectively; *ŋ*_B_ is the backscattering efficiency and σ is the depth of the layer in which the energy of the e-beam is deposited, which have been given; a, b are the scattering coefficients of the electron beam at the radius of metal vapors, and the values of these two values have been given by the above fitting results; *r*_0_ is the radius of focus point, which is set to 0.25 mm according to the relevant literature.

## 4. Results and Discussion

### 4.1. Keyhole Evolution Process

**Figure 4:**
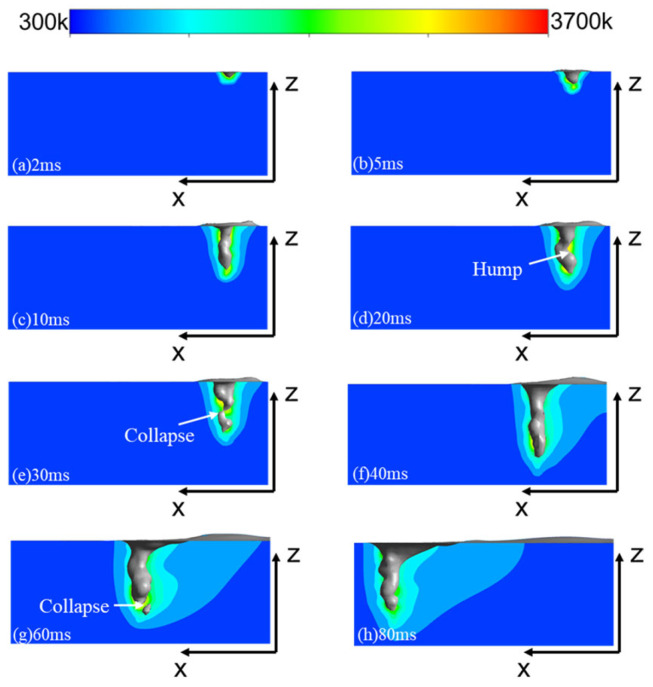
Evolutions of the keyhole profile and workpiece temperature distributions. gray color represents keyhole: (**a**) 1 ms; (**b**) 5 ms; (**c**) 10 ms; (**d**) 20 ms; (**e**) 30 ms; (**f**) 40 ms; (**g**) 60 ms; (**h**) 80 ms.

The evolution of the electron beam welding keyhole and the distribution of the temperature field are illustrated in Figure 4. The temperature field indicates that the melting region is narrow at the front of keyhole and wider at the back, with relatively dense isotherms observed in the front area. The metal at the leading edge of the melt pool continuously absorbs heat, resulting in melting and formation of the melt pool. Conversely, the temperature of the metal at the rear wall of the melt pool gradually decreases, leading to solidification and ultimately forming a weld.

Observing the evolution process of the keyhole, it is noted that, during the initial loading phase of the electron beam heat source, a period of heat accumulation occurs without the formation of a keyhole. Over time, keyholes gradually emerge due to recoil pressure. As illustrated in Figure 4a–c, the early stages of keyhole formation exhibit a more regular shape and clearer outlines. As the depth of the keyhole increases, it becomes increasingly unstable and develops humps under influences such as recoil pressure, surface tension, and hydrostatic pressure, as depicted in Figure 4d. The action of Marangoni convection causes these humps to move along the surface of the keyhole, leading to fluctuations in its walls. When these fluctuations become significant and as the keyhole advances forward, when the liquids on the front and rear walls of the keyhole make contact with each other, it will result in the collapse of the keyhole; this phenomenon is shown in Figure 4e,g.

### 4.2. Melt Flow Patterns of EBW

**Figure 5:**
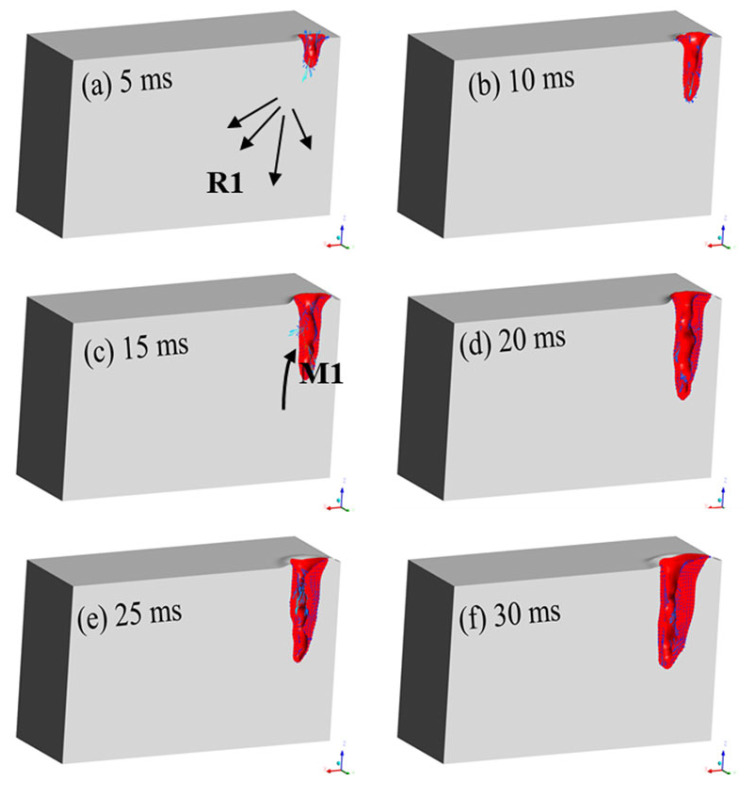
The vector evolutions in the molten pool: (**a**) 5 ms; (**b**) 10 ms; (**c**) 20 ms; (**d**) 40 ms; (**e**) 45 ms; (**f**) 70 ms.

The liquid flow behavior in the melt pool is shown in Figure 5. Initially, under the influence of recoil pressure, the liquid metal flows outward in the R1 direction (Figure 5a). As the depth of the molten pool increases, an upward M1 flow is generated due to Marangoni convection (Figure 5b). Eventually, this upward flow is impeded at the rear of the molten pool, resulting in a clockwise S1 circulation pattern (Figure 5c). When the depth of the keyhole stabilizes, liquid metal at its lower portion flows towards the rear of the molten pool as a consequence of recoil pressure (Figure 5d) and, upon obstruction, a counterclockwise S2 circulation forms beneath this region (Figure 5e,f). These two circulations transport some liquid metal toward areas near the keyhole, inducing fluctuations in its size while also conveying part of it to the rear wall of the molten pool, thereby elongating it. Due to these fluctuations in keyhole dimensions, smaller circulations emerge as illustrated in Figure 5d–f.

**Figure 6:**
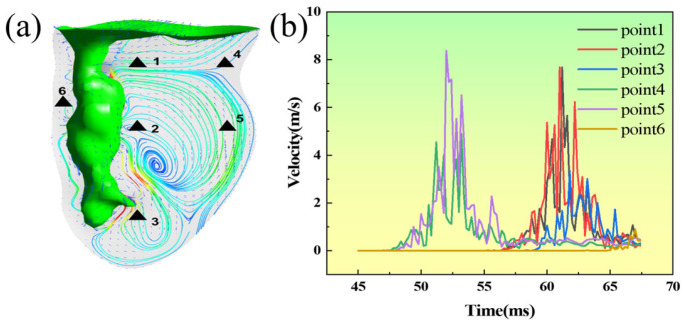
Calculated velocity evolution curves within the molten pool at 68 ms. (**a**) The liquid flow trajectory lines. (**b**) Flow velocities at six distinct points.

As illustrated in Figure 6a, the liquid flow trajectory lines within the molten pool at 68 ms are presented. The flow velocities at six distinct points within the molten pool were analyzed separately, and the results are depicted in Figure 6b. As the welding process progresses, it is observed that, the closer the liquid is to the keyhole wall, the greater its flow velocity becomes. Conversely, as the keyhole advances forward, there is a gradual reduction in flow velocity. The liquid flow in front of the keyhole remains relatively stable (P6), while faster flows are noted at positions behind the molten pool (P1 and P2). The stable flow in front of the keyhole (P6) acts as a steady inflow boundary for the rear circulation, reducing numerical fluctuation and ensuring reproducible keyhole collapse cycles. This stable region also limits bubble generation at the front, making the rear flow the dominant area for porosity formation. Additionally, significant fluctuations in flow velocity occur at higher regions of the molten pool (P1 and P4), whereas lower regions exhibit less fluctuation (P3). A comparison of liquid metal flow velocities across different positions at a given instant reveal that those at the edge of the molten pool (P4 and P5) are lower than those near the keyhole (P1 and P2).

### 4.3. Process-Induced Porosity Formation and Suppression

#### 4.3.1. Generation of Porosity Defects

**Figure 7:**
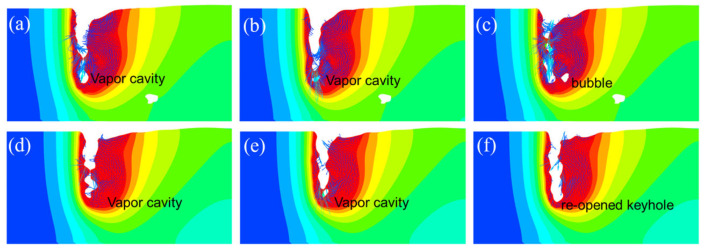
The formation process of bubbles on molten pool. (**a**) 20 ms; (**b**) 21 ms; (**c**) 22 ms; (**d**) 40 ms; (**e**) 41 ms; (**f**) 42 ms.

The generation of porosity defects within the molten pool is influenced by two primary conditions: the formation of bubbles and the inability of these bubbles to escape from the molten pool. A cavity forms beneath the collapsed keyhole, as illustrated in Figure 7a,b,e. The energy deposition from the electron beam onto the liquid above this cavity may puncture it without leading to bubble formation, as shown in Figure 7f. Conversely, a cavity that is not re-penetrated by the electron beam will be drawn towards the rear of the molten pool, resulting in bubble formation, as depicted in Figure 7c. This phenomenon creates an opportunity for porosity defects to occur.

**Figure 8:**
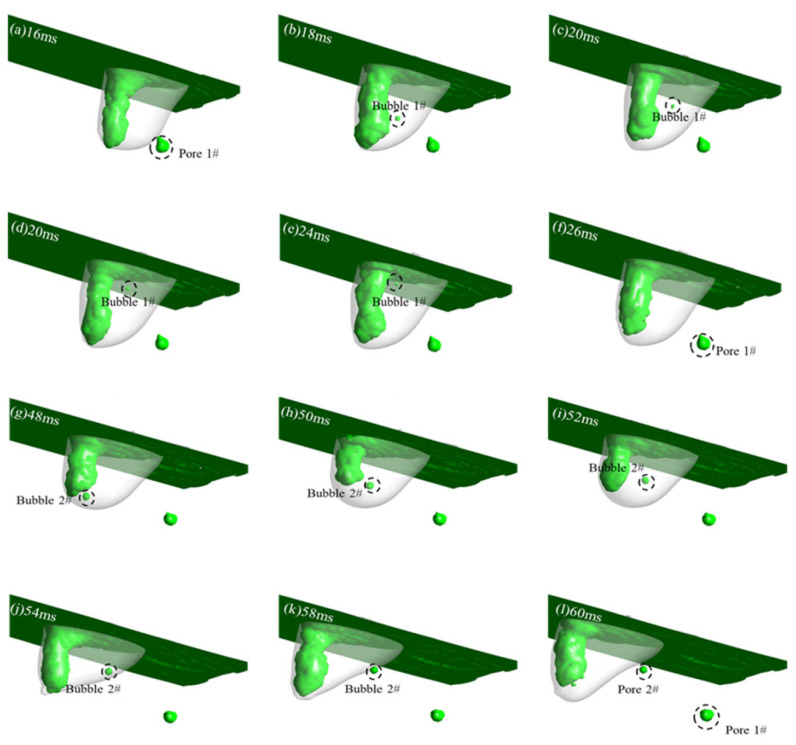
The formation process of porosity in the molten pool. (**a**) 16 ms; (**b**) 18 ms; (**c**) 20 ms; (**d**) 22 ms; (**e**) 24 ms; (**f**) 26 ms; (**g**) 30 ms; (**h**) 32 ms; (**i**) 34 ms; (**j**) 36 ms; (**k**) 38 ms; (**l**) 40 ms.

As illustrated in Figure 8, the simulation effectively captures the three-dimensional flow process of two bubbles following their formation. The gray transparent area represents the melt pool. It is observed that both bubbles are generated at the lower part of the keyhole; Bubble1# is formed at 18 ms, as depicted in Figure 8b, and escapes from the surface of the molten pool at 26 ms without resulting in a blowhole defect, as shown in Figure 8f. In contrast, Bubble2# is generated at 30 ms and reaches the gas–liquid interface by 36 ms but does not continue to flow, as indicated in Figure 8j. Ultimately, it remains within the molten pool and contributes to porosity defects, as demonstrated in Figure 8i. In addition, observing the size of the two bubbles, it can be found that the larger bubbles are less likely to escape from the melt pool, which may be due to the larger size of the bubbles by the larger liquid viscous force.

#### 4.3.2. Effect of Welding Parameters on Porosity Defects

**Figure 9:**
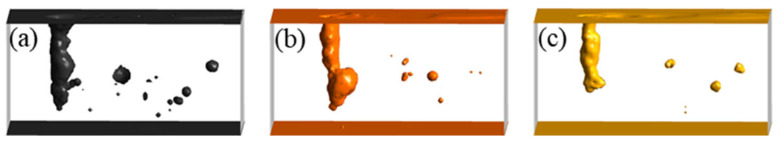
The porosity defects in the molten pool. (**a**) 35 mA, 1200 mm/min; (**b**) 45 mA, 1600 mm/min; (**c**) 60 mA, 2000 mm/min.

The porosity obtained from numerical simulations with different welding parameters is shown in Figure 9. It can be noticed that, with the increase in power and speed, the porosity defects are significantly reduced. This finding aligns with results obtained from X-ray images [9].

**Figure 10:**
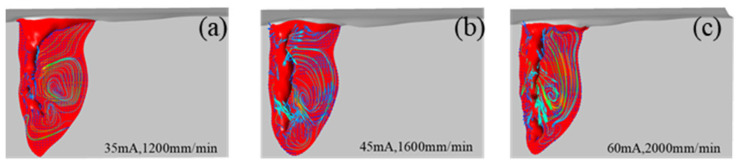
The streamlines and 3D keyhole shapes at different welding parameters: (**a**) 35 mA, 1200 mm/min; (**b**) 45 mA, 1600 mm/min; (**c**) 60 mA, 2000 mm/min.

This phenomenon can be explained in terms of both keyhole stability and melt pool flow behavior. Figure 10 depicts the flow field distribution of the molten pool intercepted at a consistent position. The molten pool exhibits an upper counterclockwise vortex and a lower clockwise vortex. In the investigated welding parameter window (35–60 mA current and 1200–2000 mm/min welding speed), both vortices gradually diminish with the increase in welding speed and power. At lower welding parameters, these two eddies converge towards the center of the keyhole. As welding parameters increase further, their convergence shifts closer to the upper region of the keyhole. With continued acceleration, the point of convergence approaches near-surface levels within the molten pool.

**Figure 11:**
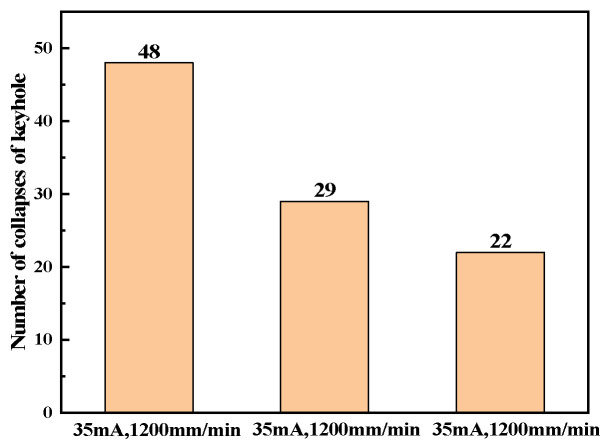
Number of keyhole collapses for different welding parameters.

**Figure 12:**
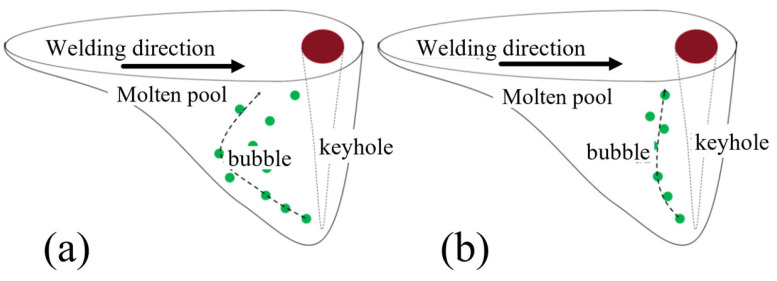
Schematic diagram of bubble flow trajectory: (**a**) high speed; (**b**) low speed.

The more complex flow system with small parameters has two effects. On the one hand, the complex circulation exacerbates the disturbance to the keyhole wall, which seriously affects the stability of the keyhole; the statistically obtained number of keyhole collapses with different parameters is shown in Figure 11. The higher the number of keyhole collapses, the higher the probability of generating bubbles. On the other hand, the presence of complex eddy currents in the melt pool hinders the upward floating of the bubbles, while the stable upward flow under the large parameter is obviously more helpful for the bubbles to escape from the surface of the melt pool, as shown in Figure 12, which is a schematic diagram of the movement trajectories of the bubbles under different welding speeds.

### 4.4. Comparison of Simulated and Experimental Weld Morphology

**Figure 13:**
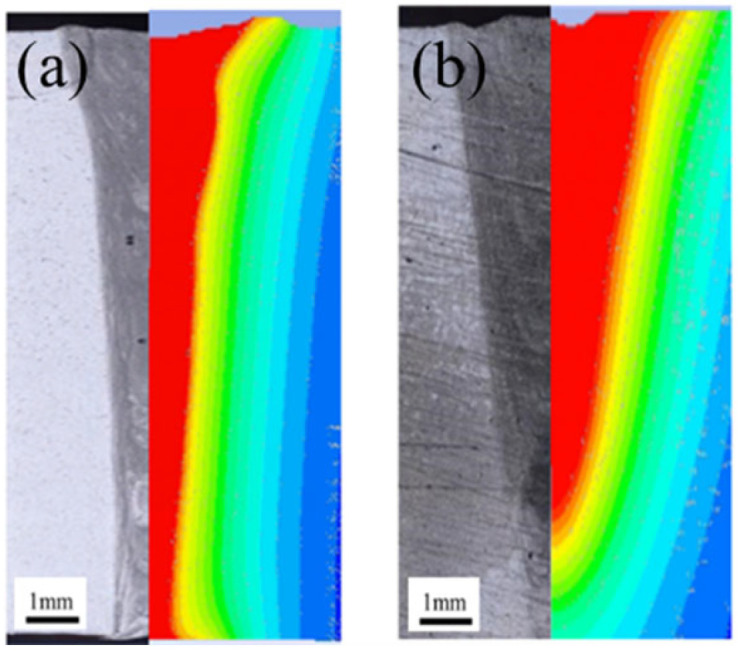
Comparison of weld pool morphology obtained by numerical simulation and experiment. (**a**) 2700 w, 1200 mm/min. (**b**) 2100 w, 1200 mm/min.

To validate the accuracy of the proposed heat source model and numerical framework, a direct comparison between the simulated weld cross-section and the experimentally obtained metallographic section is presented in Figure 13. The left half of each subfigure shows the macrograph of the experimental weld cross-section, while the right half displays the corresponding temperature field distribution and fusion boundary predicted by the simulation. The results show that the predicted weld geometry, including the fusion line profile, penetration depth, and weld width, are in excellent agreement with the experimental observations. The characteristic nail-head shape of the electron beam weld is accurately reproduced by the simulation, as well as the narrow deep-penetration profile typical of keyhole-mode welding. Quantitatively, the relative errors in penetration depth and weld width are less than 5%, confirming that the model effectively captures the transient energy deposition and heat transfer behavior during EBW.

**Figure 14:**
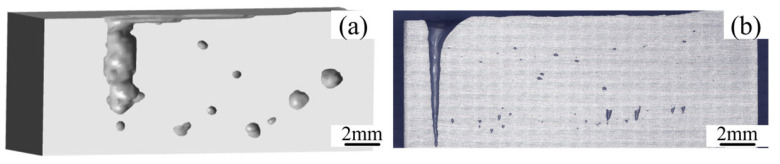
Porosity defects in the longitudinal section of aluminum alloy electron beam welding seam. (**a**) Simulated porosity defects in longitudinal section; (**b**) experimental porosity defects in longitudinal section.

Figure 14 presents the pore distribution in weld seams under both experimental welding and numerical simulation conditions. In the numerical simulation and experimental test of aluminum alloy electron beam welding, a large number of porosity defects with diverse morphologies can be clearly observed on the longitudinal section of the weld seam, and the distribution characteristics of these defects show a good consistency between simulation and experiment.

But this model ignores the mass loss induced by material evaporation and lacks an analysis of uncertainty and numerical error, which affect the accurate characterization of keyhole evolution and melt pool flow. Future work will further optimize the model to compensate for the deficiency.

## 5. Conclusions

The proposed model reveals the coupled mechanism between keyhole dynamics and porosity, providing a reliable numerical basis for quality control in EBW of 5B70 aluminum alloy. This study produced the following four primary conclusions:

(1). A three-dimensional transient model with a novel discrete tracking + vapor composite heat source is established, which accurately captures the dynamic energy coupling between electron beam and keyhole wall.

(2). Process-induced porosity is jointly controlled by keyhole instability and melt flow circulation. Collapsed cavities not re-penetrated by the beam form bubbles; large bubbles are hard to escape due to high viscous drag.

(3). Two counter-rotating circulations exist in the melt pool, converging toward the keyhole and hindering bubble escape.

(4). Increasing welding power and speed stabilizes the keyhole, weakens circulations, and reduces porosity.

## Figures and Tables

**Table 1:** Material properties of 5B70 aluminum alloy.

Physical Property	Value
Density of solid phase, kg/m^3^	2630
Density of liquid phase, kg/m^3^	2400
Latent heat of fusion, J/kg	397,000
Latent heat of evaporation, J/kg	10,800,000
Specific heat of solid phase, J/kg K	940
Surface tension at 902 K, N/m	0.81
Specific heat of liquid phase, J/kg K	1180
Boiling point, K	2720
Solidus temperature, K	833
Liquidus temperature, K	905
Ambient temperature, K	300
Gravitational acceleration, J/mol K	9.81
Universal gas constant, J/mol K	8.31
Thermal conductivity of solid phase, W/m K	243
Thermal conductivity of liquid phase, W/m K	138
Temperature coefficient of surface tension, N/m K	−0.00092

## Data Availability

The original contributions presented in the study are included in the article, further inquiries can be directed to the corresponding author.
